# First experience of hemadsorption with HA60 cartridges in newborns and small infants with septic shock: a case series

**DOI:** 10.3389/fped.2026.1769731

**Published:** 2026-06-05

**Authors:** Gabriella Bottari, Cristina Maccarrone, Antonio Torelli, Matteo Di Nardo, Anna Teresa Mazzeo, Corrado Cecchetti, Isabella Guzzo

**Affiliations:** 1 Pediatric Intensive Care Unit, Ospedale Pediatrico Bambino Gesù (IRCSS), Rome, Italy; 2 Anesthesia and Intensive Care Department of Human Pathology, University of Messina, Messina, Italy; 3 School of Pediatrics, University of “Tor Vergata”, Rome, Italy; 4 Division of Nephrology and Dialysis, Bambino Gesù Children’s Hospital, Rome, Italy

**Keywords:** cytokines, hemoadsorption, infants, newborns, pediatric intensive care, pediatric septic shock

## Abstract

**Background:**

Blood purification techniques are rarely used in low-weight pediatric patients because extracorporeal circuits and devices designed for adults may expose neonates and infants to significant adverse events. Recently, hemoadsorption cartridges with low priming volumes (Jafron®) have become available.

**Case description:**

We report a descriptive case series of four pediatric patients treated with hemoadsorption using HA60 cartridges, including two neonates (2.8 kg and 3.5 kg) and two infants (7 kg and 10 kg). All patients were admitted to the PICU with septic shock and received standard therapy, including broad-spectrum antibiotics and vasoactive agents. Two of the four patients required extracorporeal membrane oxygenation (ECMO) because of severe respiratory and circulatory failure. In all patients, continuous renal replacement therapy (CRRT) was initiated for acute kidney injury and/or fluid overload, and HA60 hemoadsorption was added. Clinical and laboratory parameters, together with vasoactive drug requirements, were recorded from the initiation of hemoadsorption until the end of treatment. Three of the four patients survived.

**Discussion and conclusions:**

Our findings are limited to describing the feasibility and safety of HA60 hemoadsorption in neonates and small infants with septic shock, as well as the logistical aspects associated with the use of these novel extracorporeal technologies, rather than demonstrating clinical efficacy. No major adverse events were observed. Within this perspective, the fatal outcome observed in Case 1 was considered unrelated to the hemoadsorption procedure itself. Given the descriptive nature of the study and the presence of multiple concomitant therapies, no conclusions regarding efficacy can be drawn. Larger studies are needed to confirm these preliminary observations.

## Introduction

Hemoadsorption (HA) is an extracorporeal blood purification technique aimed at enhancing the removal of solutes, cells, or pathogens that are not effectively cleared by diffusive or convective mechanisms because of their physicochemical properties (1). To date, these techniques are not recommended in current guidelines for the management of pediatric septic shock, mainly because of the lack of evidence from multicenter randomized controlled trials (2, 3). In 2024, a consensus statement on extracorporeal blood purification techniques in critically ill pediatric patients was published (4). The authors acknowledged the potential role of these therapies in critically ill children; however, they also highlighted important concerns, including the lack of devices specifically designed for pediatric patients and the absence of high-quality evidence supporting their use (4).

Over recent years, a growing body of evidence has emerged regarding the potential application of hemoadsorption in pediatric populations (5). The rationale underlying its use is that hemoadsorption may be considered in critically ill children when the expected benefits—such as modulation of inflammation, toxin removal, or organ support—are likely to outweigh the risks associated with extracorporeal circulation and procedural invasiveness (6). Nevertheless, although the applicability of these techniques has progressively improved in pediatric patients, significant concerns remain regarding their safe use in neonates and small infants, a particularly vulnerable population undergoing extracorporeal treatments (7, 8). The main technical limitations in this group are related to the increased risk of adverse events due to extracorporeal circuit volume exceeding safe thresholds, potentially leading to hypotension and hemodynamic instability, as well as hemodilution resulting in thrombocytopenia and anemia (9).

CytoSorb (CytoSorbents, USA) is a non-selective hemoadsorption cartridge composed of highly porous styrene–divinylbenzene copolymer beads coated with polyvinylpyrrolidone (PVP), enabling the adsorption of molecules up to 60 kDa (e.g., cytokines, myoglobin, bilirubin, and drugs). The PVP coating enhances biocompatibility and reduces the adsorption of non-target molecules. CytoSorb has a priming volume of 120 mL and is typically used for up to 24 h. To date, it has been the most widely used hemoadsorption device in pediatric settings, with promising results reported in children (5, 9, 10). Reported benefits include its use in septic shock and hyperinflammatory syndromes through immunomodulation of the cytokine storm responsible for shock and multiorgan dysfunction, as well as in liver failure and rhabdomyolysis because of its ability to remove bilirubin, bile acids, and myoglobin (9).

Recently, cartridges specifically designed for low-weight patients have been introduced (Jafron, China). These devices are smaller than conventional cartridges and are characterized by reduced extracorporeal blood volume. The HA60 cartridge, designed for pediatric use, is a non-selective device composed of highly porous styrene–divinylbenzene copolymer beads capable of adsorbing molecules up to 60 kDa, with chemically treated surfaces to enhance biocompatibility. It has a sorbent volume of 65 mL, a reduced priming volume, and is typically used for 6–12 h per day. In pediatric populations, only a limited number of studies have been published, suggesting a potentially beneficial role for these devices in smaller patients (11, 12).

In this report, we describe a case series of four patients focusing on the use of HA60 hemoadsorption in newborns and small infants with septic shock, with particular attention to the feasibility and safety of this approach in a highly vulnerable population.

## Case description

This study is a descriptive case series involving four pediatric patients admitted to the Pediatric Intensive Care Unit (PICU) of Bambino Gesù Children's Hospital in Rome, Italy. The series included two newborns and two young infants affected by refractory septic shock unresponsive to standard therapy who were treated with HA60 cartridges as adjunctive therapy. The cases were consecutively enrolled between 2022 and 2024. Written informed consent for participation in this study and for publication was obtained from the patients’ legal guardians or next of kin.

**Figure 1:**
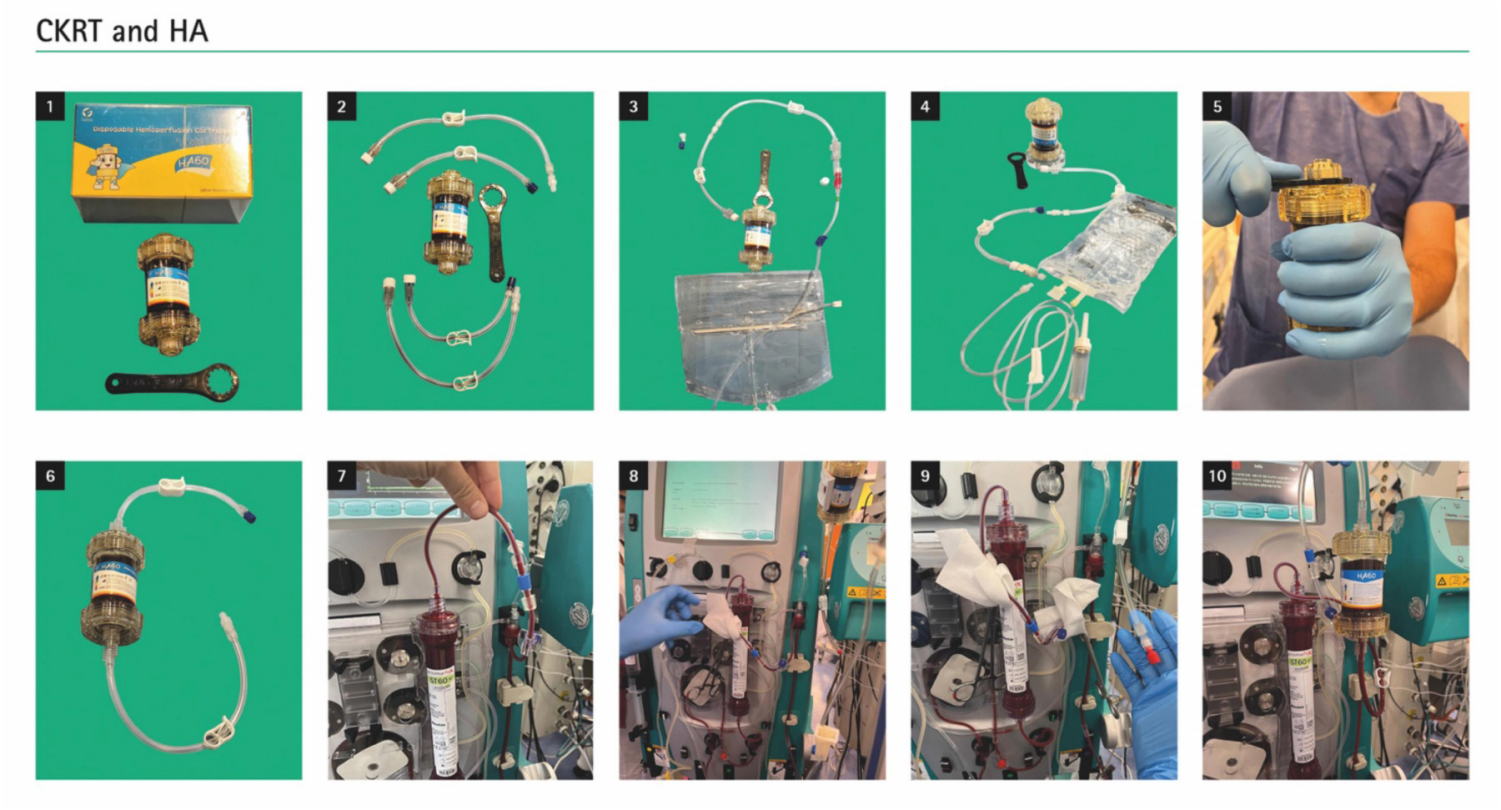
Graphic illustration of the priming and mounting of the HA60 cartridge in series with a dialysis hemofilter.

**Figure 2:**
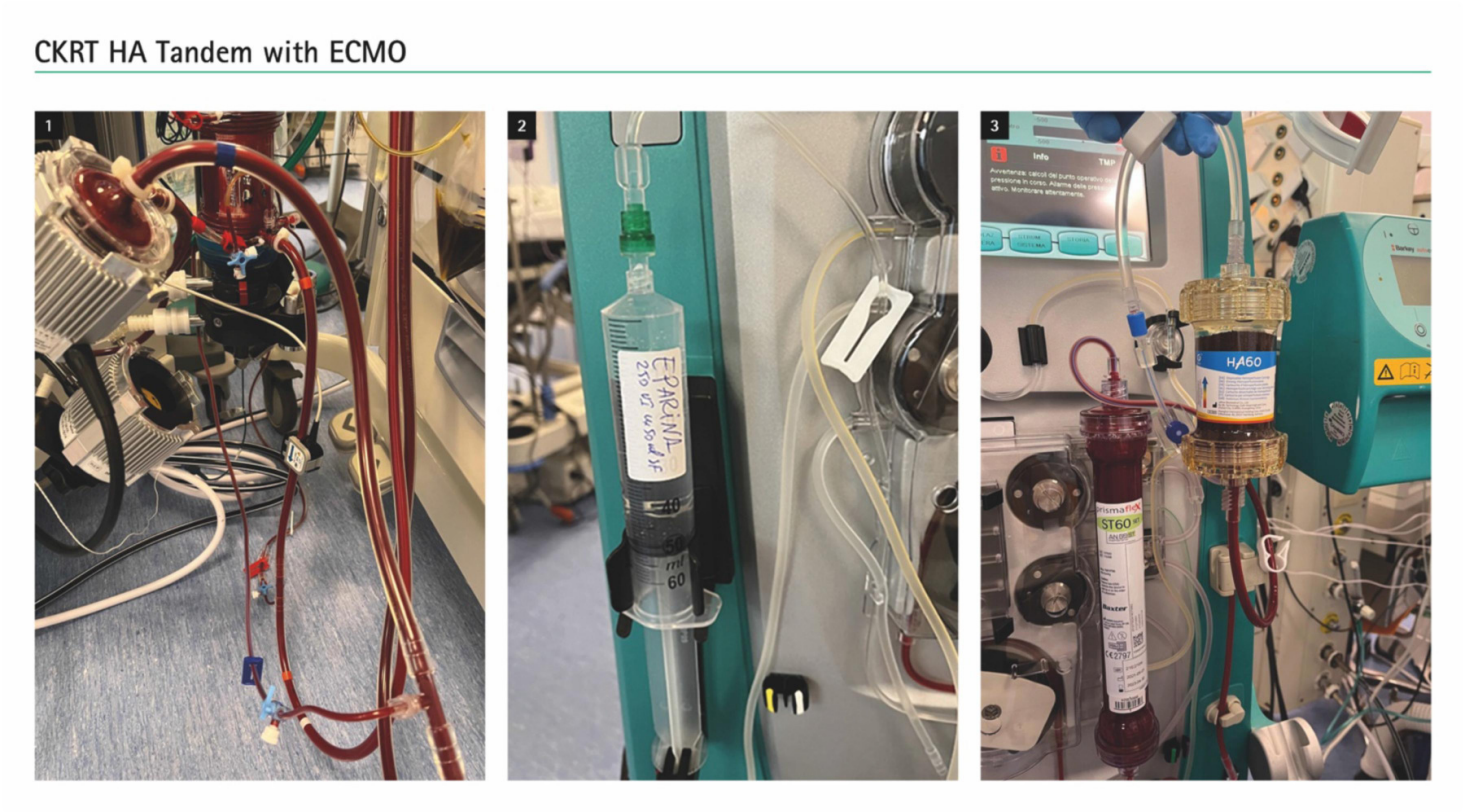
Graphic illustration of the mounting of the HA60 cartridge in series with a dialysis hemofilter and in tandem with ECMO.

A hemodialysis catheter was inserted into a central vein (internal jugular or femoral), according to patient size and clinical characteristics. Continuous renal replacement therapy (CRRT) was performed using standard hemofilters (polyarylethersulfone or AN69ST) combined with HA60 in continuous venovenous hemodiafiltration (CVVHDF) modality, with pre-filter replacement fluid administration, an effluent dose of 2000mL/h/1.73 m^2^, and a blood flow rate ranging from 20 to 60 mL/min. The HA60 cartridge was inserted into the CRRT circuit in series with the hemofilter in a post-filter position (Figure 1). The CRRT circuit and HA60 cartridge were primed with saline solution and packed red blood cells. Anticoagulation was managed using regional citrate anticoagulation. In children also supported with extracorporeal membrane oxygenation (ECMO), systemic anticoagulation was achieved with bivalirudin, while regional citrate anticoagulation was maintained within the CRRT circuit. Hemoadsorption therapy was continued according to the clinical course and laboratory surrogate markers (including lactate levels, metabolic status, and pH), following a therapeutic schedule of 6–12 h/day for 2 or more days, as recommended by the manufacturer. In patients receiving ECMO support, the CRRT access line was placed post-oxygenator and the return line post-pump (Figure 2).

Treatment initiation was guided by clinical evolution during an observational period of up to 6 h under standard therapy and was based solely on the attending physicians’ clinical judgment. In particular, hemoadsorption was initiated in patients showing signs of clinical deterioration, including increasing lactate levels, escalating vasopressor requirements, the need for additional inotropic support, and/or evidence of sepsis-associated myocardial dysfunction.

Adverse events were evaluated retrospectively. Specifically, we reviewed the occurrence of severe hypotensive episodes (according to age-adjusted mean arterial pressure values) during connection to the CRRT-HA circuit, as well as severe hemodilution, anemia, or thrombocytopenia occurring during hemoadsorption and CRRT treatment.

## Case 1

A two-month-old male infant (body weight 2.8 kg, height 48 cm) was admitted to the PICU with abdominal septic shock secondary to necrotizing enterocolitis (NEC), previously treated with bowel resection and protective ileostomy. Following surgery, the patient developed severe hemodynamic instability associated with severe myocardial dysfunction (ejection fraction 20%–25% on two-dimensional echocardiography), requiring veno-arterial extracorporeal membrane oxygenation (VA-ECMO) before transfer to our PICU.

Six hours after PICU admission, despite standard therapy including meropenem, vancomycin, and metronidazole, the patient developed acute kidney injury (AKI) characterized by anuria, metabolic acidosis, and hyperlactatemia. Continuous renal replacement therapy (CRRT) was therefore initiated in tandem with VA-ECMO. Considering the severe inflammatory state associated with multiorgan failure, we decided to combine CRRT with hemoadsorption using an HA60 cartridge immediately after CRRT initiation.

Two hemoadsorption cycles were performed over 48 consecutive hours: the first lasted 8 h and the second 6 h. No hemoadsorption device-related complications were observed.

**Figure 3:**
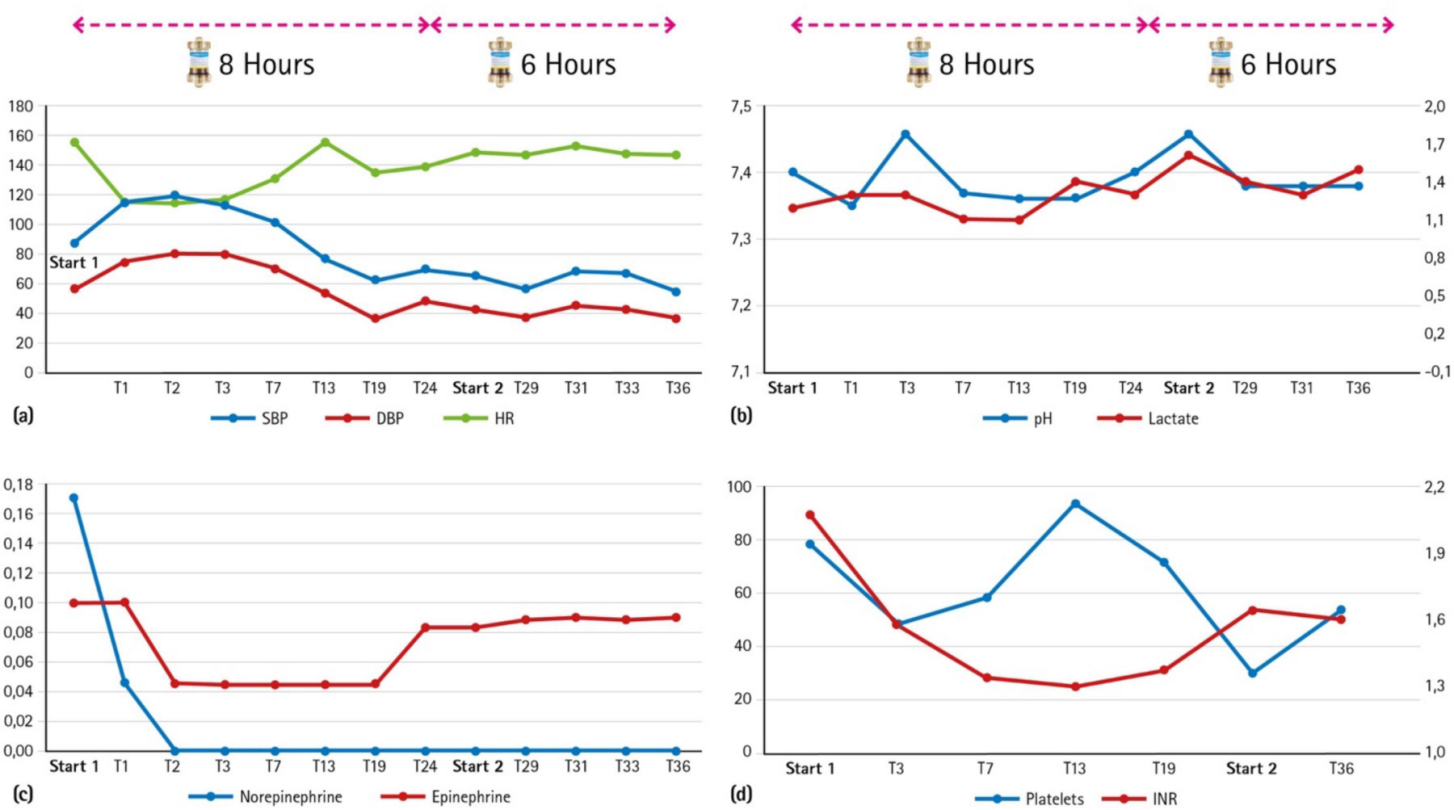
These figures describe the temporal trends of the data collected from the beginning of the first HA60 cycle to the end of the last cycle, once the cartridge has been removed from the extracorporeal circuit: **(a)** hemodyanmic parameters (SBP, systolic blood pressure; DBP, diastolic blood pressure; HR, heart rate), **(b)** arterial blood gas analysis parameters: pH and Lactates (Lac, lactate), **(c)** vasoactive drugs doses (epinephrine and nor-epinephrine) and coagulation parameters: platelets count and INR (INR, international normalized ratio; PLT, platelets count).

Figure 3 reports time-course of hemodynamic parameters (a), inotropes and vasopressors doses (b), metabolic values and perfusion indexes time-course (c) and coagulation parameters (d).

Lactate levels remained stable during both treatment cycles. The epinephrine dose was reduced by half (0.045 µg/kg/min at T8), and norepinephrine was discontinued before the end of the first cycle because of improvement in hemodynamic and perfusion parameters, including restoration of diuresis.

Supplementary Figure 1 shows the blood bowels perfusion after a monitoring at the end of the first cycle of HA.

During the following 24 h, the patient experienced further clinical deterioration due to persistent abdominal sepsis. Consequently, the epinephrine dose was increased again to 0.089 µg/kg/min and remained stable until the end of the second treatment cycle. Unfortunately, three days later, the patient died because of extensive bowel necrosis, refractory septic shock, and disseminated intravascular coagulation (DIC). Notably, microbiological cultures failed to isolate any microorganisms from the collected samples.

Table 1 summarizes the data collected at the initiation of hemoadsorption treatment (T0) and at the end of the final treatment cycle, after removal of the cartridge from the extracorporeal circuit (Tend).

**Table 1:** Reports the data collected at beginning of hemoadosption treatment **(T0)** and at the end of the last cycle, once the cartridge has been removed from the extracorporeal circuit **(tend).**

Vital signs, laboratory values, and vasopressor dosage	Case 1		Case 2		Case 3		Case 4	
	T_0_	T_end_	T_0_	T_end_	T_0_	T_end_	T_0_	T_end_
SBP (mmHg)	88	67	52	71	124	74	87	106
DBP (mmHg)	55	42	27	55	86	61	41	62
HR (bpm)	155	147	178	152	133	159	159	81
Epinephrine (y/kg/min)	0.10	0.08	0.21	0.07	0.07	0.10	0.08	0.03
Norepinephrine (y/kg/min)	0.17	0	0.13	0	0.05	0.21	0.3	0
Lactate (mmol/L)	1.2	1.5	13.8	2.3	1	1.7	1.9	1.3
INR	2.08	1.61	2.33	1.4	1.3	1.5	2.34	1.45
PLT (mm^3^/mL)	79.000	53.000	57.000	40,000	31.000	61.000	24.000	24.000
C-reactive protein (mg/dL)	21	14.6	2.1	16.9	28.5	21	24.1	12.8
Procalcitonin (ng/mL)	4.1	1.3	62.7	27.5	10	3.6	47.3	43.3

SBP, systolic blood pressure; DBP, diastolic blood pressure; HR, heart rate; INR, international normalized ratio; PLT, platelets count.

## Case 2

A one-day-old female newborn (body weight 3.5 kg, height 55 cm), born at term (40 weeks of gestational age), was admitted to the PICU for respiratory failure secondary to intrapartum meconium aspiration syndrome associated with severe pulmonary hypertension requiring support with veno-arterial extracorporeal membrane oxygenation (VA-ECMO). The patient received broad-spectrum antibiotic therapy with amikacin and meropenem; however, she rapidly developed septic shock and sepsis-associated acute kidney injury (S-AKI) within six hours.

Continuous renal replacement therapy (CRRT) was initiated in tandem with VA-ECMO, and hemoadsorption using an HA60 cartridge was performed in series with the CRRT hemofilter. Two hemoadsorption cycles were carried out. The first cycle lasted only 4 h and was interrupted because of technical issues related to high pressures transmitted from the ECMO circuit to the CRRT circuit. The second cycle lasted 12 h. No hemoadsorption device-related complications were observed.

**Figure 4:**
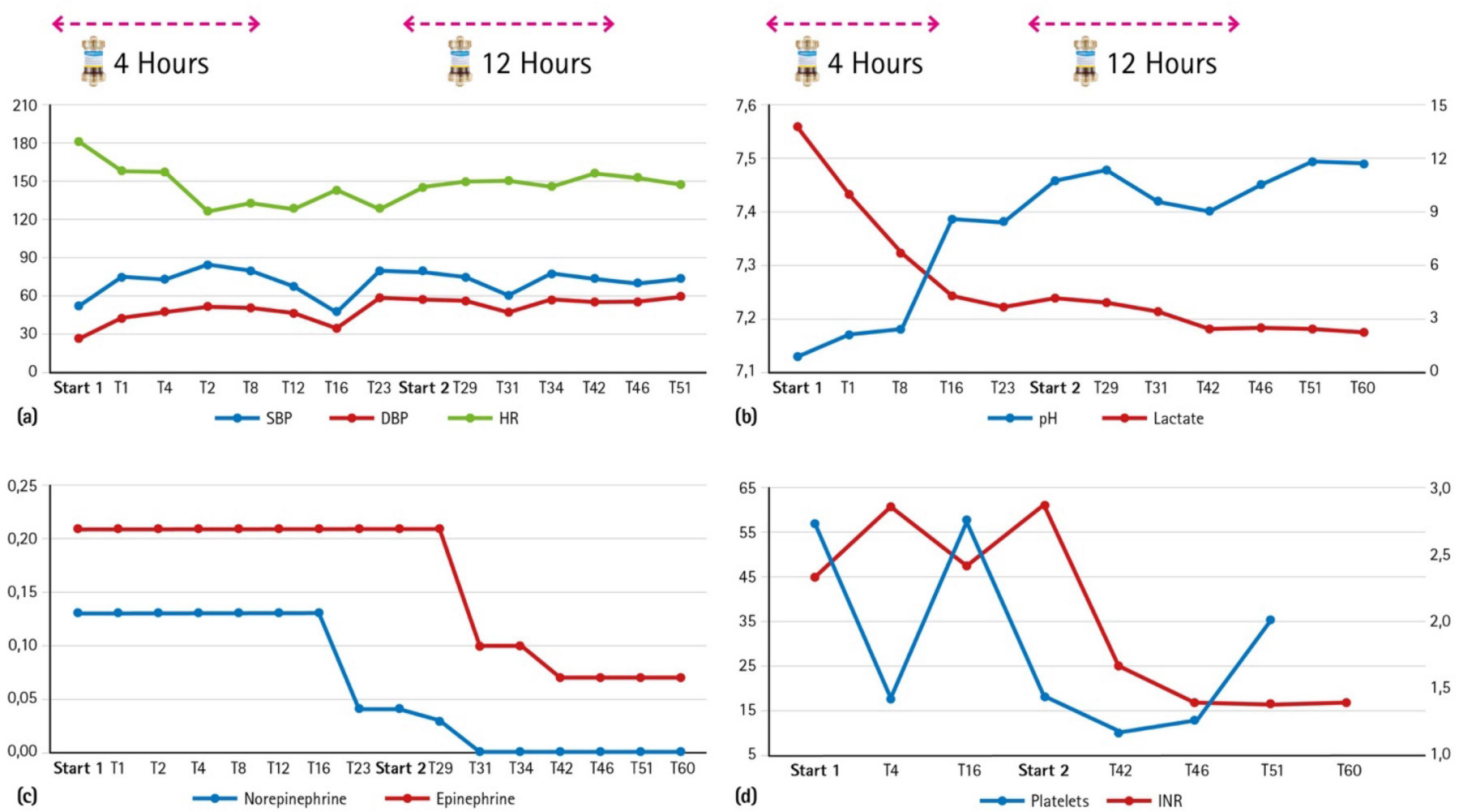
These figures describe the temporal trends of the data collected from the beginning of the first HA60 cycle to the end of the last cycle, once the cartridge has been removed from the extracorporeal circuit: **(a)** hemodyanmic parameters (SBP, systolic blood pressure; DBP, diastolic blood pressure; HR, heart rate), **(b)** arterial blood gas analysis parameters: pH and lactates (Lac, lactate), **(c)** vasoactive drugs doses (epinephrine and nor-epinephrine) and coagulation parameters: platelets count and INR (INR, international normalized ratio; PLT, platelets count).

Figure 4 reports time-course of hemodynamic parameters (a), inotropes and vasopressors doses (b), metabolic values and perfusion indexes time-course (c) and coagulation parameters (d).

Escherichia coli was isolated from the tracheal aspirate culture, and the antibiogram demonstrated good sensitivity to meropenem. By the end of the second hemoadsorption cycle, norepinephrine had been discontinued and the epinephrine dose had been markedly reduced. Lactate levels progressively normalized over time.

Supplementary Figure 2 illustrates the evolution of acute lung injury on chest x-ray before and after 48 h of HA60 hemoadsorption treatment. Table 1 summarizes the data collected at the initiation of hemoadsorption therapy (T0) and at the end of the final treatment cycle, after removal of the cartridge from the extracorporeal circuit (Tend).

Continuous kidney replacement therapy (CKRT) was discontinued together with hemoadsorption after 48 h. No adverse events were observed. ECMO support was discontinued after 7 days, and the patient was discharged from the PICU after 9 days.

## Case 3

A three-month-old male infant (body weight 7 kg, height 55 cm), born at term (40 weeks of gestational age), was admitted to the PICU with severe cytopenia, polyserositis, and acute kidney injury (AKI), later associated with suspected hemophagocytic lymphohistiocytosis (HLH). Since admission, the patient had received broad-spectrum empirical antimicrobial therapy including gentamicin, meropenem, sulfamethoxazole/trimethoprim, caspofungin, and acyclovir. Significant pericardial and ascitic effusions were drained. Fluid overload and impaired renal function required initiation of continuous renal replacement therapy (CRRT).

Two days later, despite maximal supportive therapy, the patient's clinical condition deteriorated, likely because of septic shock, as documented by pancytopenia, worsening inflammatory markers, increasing vasoactive drug requirements, and positive blood cultures for *Pseudomonas aeruginosa* and *Acinetobacter baumannii*. Antimicrobial therapy was therefore adjusted to ceftazidime/avibactam, cefiderocol, and colistin. To support the removal of pro-inflammatory cytokines in the setting of septic shock, an HA60 cartridge was added to the CRRT circuit.

Two hemoadsorption cycles were performed over 48 consecutive hours: the first lasted 9 h and the second 11 h. No hemoadsorption device-related complications were observed.

**Figure 5:**
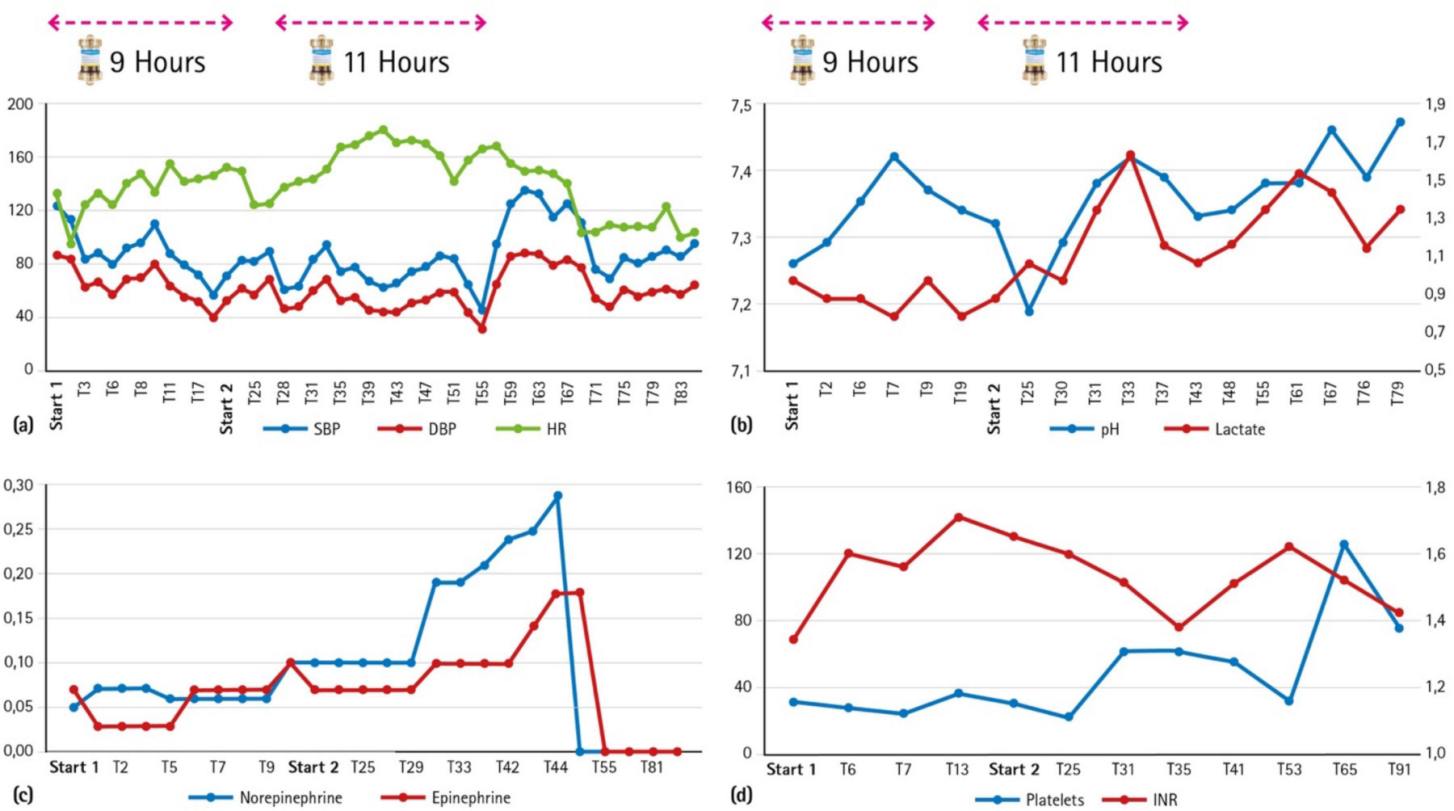
These figures describe the temporal trends of the data collected from the beginning of the first HA60 cycle to the end of the last cycle, once the cartridge has been removed from the extracorporeal circuit: **(a)** hemodyanmic parameters (SBP, systolic blood pressure; DBP, diastolic blood pressure; HR, heart rate), **(b)** arterial blood gas analysis parameters: pH and lactates (Lac, lactate), **(c)** vasoactive drugs doses (epinephrine and nor-epinephrine) and coagulation parameters: platelets count and INR (INR, international normalized ratio; PLT, platelets count).

Figure 5 illustrates the time course of hemodynamic parameters (a), inotropic and vasopressor support (b), metabolic and perfusion parameters (c), and coagulation parameters (d). Table 1 summarizes the data collected at the initiation of hemoadsorption treatment (T0) and at the end of the final treatment cycle, after removal of the cartridge from the extracorporeal circuit (Tend).

Three days later, the patient showed significant clinical improvement: both epinephrine and norepinephrine were permanently discontinued, CRRT was continued for an additional 8 days before definitive discontinuation, and the patient was extubated after 10 days. The overall clinical condition progressively improved during the PICU stay, and the infant was discharged from the PICU 11 days after completion of HA60 therapy.

## Case 4

A seven-month-old female infant (body weight 10 kg, height 75 cm), born at term (39 weeks of gestational age), was admitted to the PICU for respiratory failure associated with bronchiolitis caused by Respiratory Syncytial Virus (RSV), Parainfluenza virus, and Rhino/Enterovirus infection. Initially, she was managed with helmet continuous positive airway pressure (cPAP); however, after several days, her respiratory condition worsened, requiring invasive mechanical ventilation. Broad-spectrum antimicrobial therapy with ceftriaxone, ribavirin, and immunoglobulins was initiated.

Microbiological and culture tests subsequently revealed pulmonary infections caused by *Haemophilus influenzae*, *Moraxella catarrhalis*, *Streptococcus pneumoniae*, and *Pseudomonas aeruginosa*. Three days later, *Pseudomonas aeruginosa* was also isolated from blood cultures, prompting targeted antimicrobial therapy initially with ceftolozane/tazobactam and amikacin, and subsequently with cefiderocol, levofloxacin, and fosfomycin. In addition, detection of Cytomegalovirus (CMV) infection required initiation of ganciclovir therapy.

Despite antimicrobial treatment, the patient developed septic shock requiring vasoactive support for severe hemodynamic instability. The onset of acute kidney injury (AKI), fluid overload, and significant abdominal effusion led to the initiation of continuous renal replacement therapy (CRRT).

An HA60 cartridge was added to the CRRT circuit, and three hemoadsorption cycles were performed. The first cycle lasted 9 h, whereas the second and third cycles lasted 4 h each and were prematurely interrupted because of venous catheter malfunction, necessitating replacement of the central venous catheter. Between the second and third cycles, a new cartridge was used for 30 min; however, treatment was interrupted because of coagulation within the extracorporeal circuit. Despite these technical issues related to vascular access management, no hemoadsorption device-related complications were observed.

**Figure 6:**
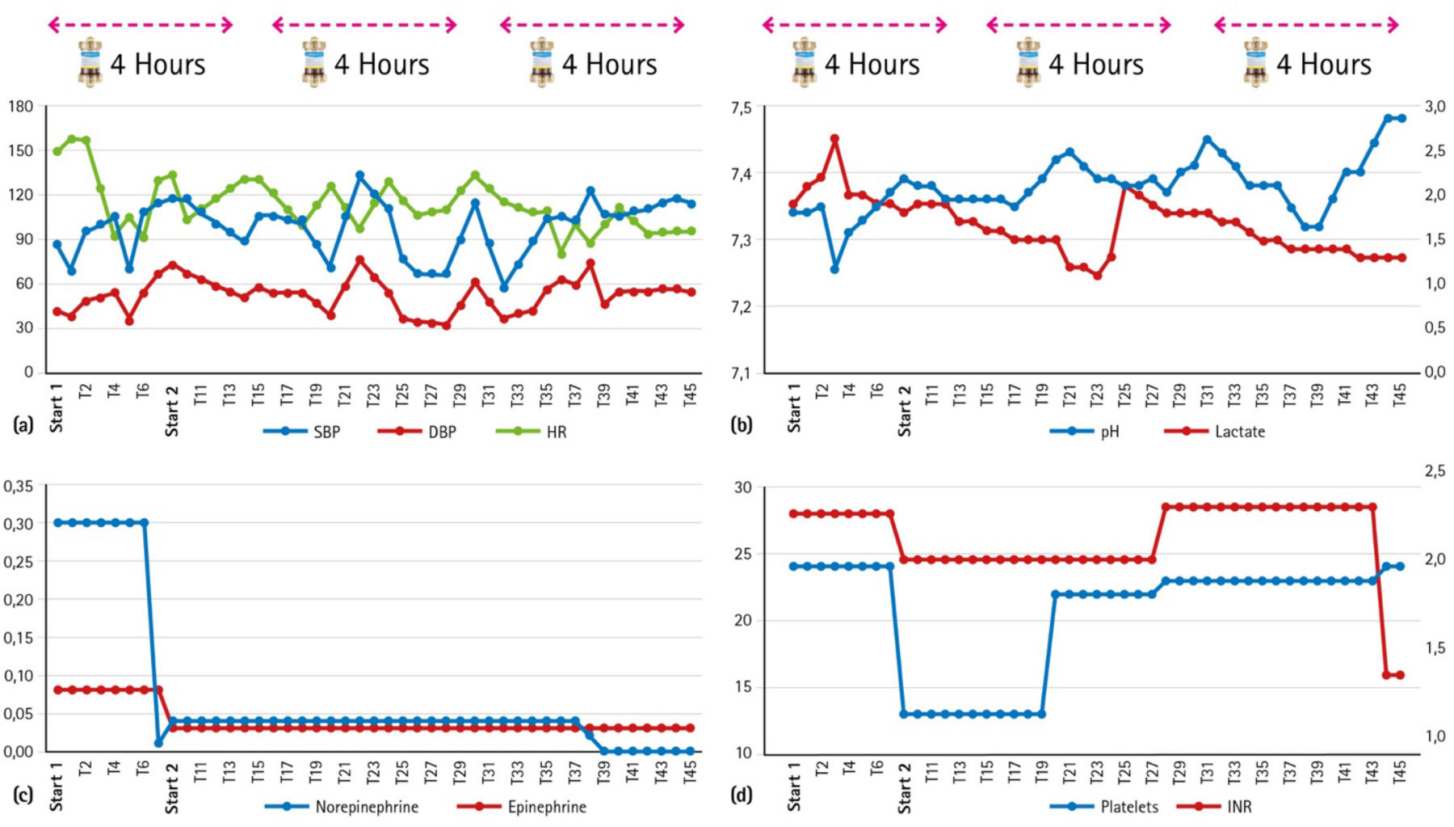
These figures describe the temporal trends of the data collected from the beginning of the first HA60 cycle to the end of the last cycle, once the cartridge has been removed from the extracorporeal circuit: **(a)** hemodyanmic parameters (SBP, systolic blood pressure; DBP, diastolic blood pressure; HR, heart rate), **(b)** arterial blood gas analysis parameters: pH and Lactates (Lac, lactate), **(c)** vasoactive drugs doses (epinephrine and nor-epinephrine) and coagulation parameters: platelets count and INR (INR, international normalized ratio; PLT, platelets count).

Figure 6 illustrates the time course of hemodynamic parameters (a), inotropic and vasopressor support (b), metabolic and perfusion parameters (c), and coagulation parameters (d). Table 1 summarizes the data collected at the initiation of hemoadsorption treatment (T0) and at the end of the final treatment cycle, after removal of the cartridge from the extracorporeal circuit (Tend). After 48 h of HA60 hemoadsorption treatment, continuous renal replacement therapy (CRRT) was continued for an additional 5 days before being definitively discontinued. The patient's overall clinical condition progressively improved during the PICU stay, follow-up culture tests became negative, and antimicrobial therapy was subsequently discontinued. The infant was ultimately discharged from the PICU 25 days after completion of HA60 therapy.

## Discussion

We report a descriptive clinical experience involving four low-weight critically ill pediatric patients treated with HA60 hemoadsorption combined with CRRT and, in selected cases, ECMO support for septic shock. In our experience, hemoadsorption was used as rescue therapy in patients presenting with septic shock, multiple organ dysfunction, and hyperinflammatory states characterized by cytokine storm. However, given the descriptive nature of this case series and the concomitant use of multiple advanced supportive therapies, including ECMO and CRRT, no causal relationship between hemoadsorption and the observed clinical changes can be established. Therefore, all variations in hemodynamic parameters, laboratory findings, and vasoactive drug requirements should be interpreted as descriptive temporal associations rather than treatment effects. Furthermore, because of the marked heterogeneity of the included patients in terms of age, underlying disease, infectious etiology, and extracorporeal support modalities, each case should be interpreted individually.

As these cases derive from routine clinical practice rather than protocol-driven investigations, both the initiation and duration of treatment were determined exclusively according to the treating physicians’ clinical judgment, while also considering technical and organizational aspects (e.g., preference for performing treatment cycles during daytime hours). In some cases, treatment variability was related to technical issues involving vascular access, as observed in Case 4. Table 2 provides an overview clinical characteristics, technical aspects, and outcomes of the four pediatric patients.

**Table 2:** Clinical characteristics, technical aspects, and outcomes of the four pediatric patientstreated with HA60 hemoadsorption.

Variable	Case 1	Case 2	Case 3	Case 4
Diagnosis	NEC, septic shock	MAS, pneumonia, septic shock	HLH, septic shock	Respiratory failure, septic shock
Organ failure	Gastrointestinal, cardiovascular, respiratory, renal	Cardiovascular, respiratory, renal	Cardiovascular, respiratory, renal	Cardiovascular, respiratory, renal
Microbiology	Negative cultures	Escherichia coli	Pseudomonas aeruginosa, Acinetobacter baumannii	Pseudomonas aeruginosa, Cytomegalovirus
Indication for CRRT	AKI, metabolic acidosis, fluid overload	AKI, metabolic acidosis, fluid overload	AKI, fluid overload, metabolic acidosis	AKI, metabolic acidosis, fluid overload
Indication for HA60	Adjunctive therapy for septic shock and MODS	Adjunctive therapy for septic shock and MODS	Adjunctive therapy for septic shock and hyperinflammation associated with HLH	Adjunctive therapy for septic shock
HA60 cycles	2 cycles (8 h + 6 h)	2 cycles (4 h + 12 h)	2 cycles (9 h + 11 h)	3 cycles (9 h + 4 h + 4 h)
Timing of HA60 initiation	At CRRT initiation	At CRRT initiation	48 h after CRRT initiation	At CRRT initiation
Technical setup	ECMO tandem; CVVHDF modality; AN69ST hemofilter	ECMO tandem; CVVHDF modality; AN69ST hemofilter	CVVHDF modality; AN69ST hemofilter	CVVHDF modality; AN69ST hemofilter
Complications	None	First cycle interrupted after 4 h because of high pressure within the CRRT circuit	None	Vascular access malfunction resulting in circuit clotting
Post-HA60 course (short-term outcome)	Worsening abdominal sepsis	ECMO discontinued after 7 days; CKRT discontinued after 2 days	CKRT discontinued after 8 days	CKRT discontinued after 5 days
Outcome	Death (bowel necrosis, DIC)	PICU discharge (day 9)	PICU discharge (day 11)	PICU discharge (day 25)

AKI, acute kidney injury; CKRT, continuous kidney replacement therapy; CRRT, continuous renal replacement therapy; CVVHDF, continuous venovenous hemodiafiltration; DIC, disseminated intravascular coagulation; ECMO, extracorporeal membrane oxygenation; HA, hemoadsorption; HLH, hemophagocytic lymphohistiocytosis; MAS, meconium aspiration syndrome; MODS, multiple organ dysfunction syndrome; NEC, necrotizing enterocolitis; PICU, Pediatric Intensive Care Unit.

Case 1 was characterized by culture-negative septic shock associated with necrotizing enterocolitis (NEC) and severe postoperative complications, including myocardial dysfunction, AKI, and disseminated intravascular coagulation (DIC). The complexity of the clinical picture makes it difficult to distinguish septic shock from catastrophic abdominal syndrome. Nevertheless, the absence of source control due to extensive bowel necrosis and the impossibility of further surgical intervention appear to have been the primary determinants of the fatal outcome. For this reason, the case should be considered clinically neutral with respect to the potential role of hemoadsorption. Notably, the patient remained relatively stable during HA treatment, and the clinical deterioration leading to death occurred three days after the last hemoadsorption cycle.

Case 3 was admitted to the PICU because of suspected hemophagocytic lymphohistiocytosis (HLH) and subsequently developed Gram-negative septic shock. Both HLH-associated hyperinflammation and septic shock are recognized indications for hemoadsorption as rescue therapy according to the available literature. Case 4 was initially admitted for viral bronchiolitis and subsequently developed bacterial superinfection and Cytomegalovirus (CMV) reactivation. In Cases 1 and 2, HA60 treatment was initiated simultaneously with CRRT because of fulminant septic shock associated with multiple organ failure shortly after PICU admission. In contrast, in Cases 3 and 4, HA60 was introduced later during the PICU stay because of superimposed septic shock in the context of progressive clinical deterioration.

No major device-related adverse events were retrospectively identified, including severe hypotension, thrombocytopenia, or clinically significant hemodilution during HA treatment. In Case 2, clotting of the extracorporeal circuit occurred because of elevated pressures transmitted from the ECMO circuit to the CRRT system. Similar technical concerns have been previously described by other authors, who suggested the use of dedicated pressure-controlled connection lines (11, 12). In Case 4, circuit clotting was associated with vascular access malfunction, further emphasizing the importance of achieving adequate blood flow to ensure effective extracorporeal blood purification treatment (13).

Previous reports have described the use of hemoadsorption in newborns and small infants (14–16). Two small case series (n = 8 each) reported higher mortality rates in patients weighing less than 10 kg compared with heavier children, warranting caution despite the absence of adjusted analyses (16, 17). More recently, the Cytoped multicenter observational study also identified higher mortality among children weighing less than 14 kg (5).

Several reports describing the use of HA60 during cardiopulmonary bypass and ECMO support have already been published. Although no major adverse events were reported, larger studies are still needed to confirm the safety and efficacy of these approaches. Our experience adds further descriptive evidence supporting the feasibility of hemoadsorption in newborns and small infants with septic shock and multiple organ dysfunction in highly complex clinical settings.

This study has several limitations. First, the very small sample size and the marked heterogeneity of the patients prevent any generalization of the findings. Second, no cytokine or inflammatory mediator measurements were performed, as the aim of this study was purely descriptive. Third, the absence of standardized criteria for treatment initiation and outcome assessment further limits interpretability. Finally, the concomitant use of multiple advanced therapies does not allow isolation of the specific contribution of hemoadsorption to patient outcomes.

## Conclusions

In our experience, HA with the HA60 cartridge was feasible and well tolerated in low-weight pediatric patients with septic shock and multiorgan failure. However, no conclusions regarding clinical efficacy can be drawn. Further studies are needed to better define the role, timing, and patient selection for this therapy in the pediatric population.

## Data Availability

The raw data supporting the conclusions of this article will be made available by the authors, without undue reservation.
