# Supplementary material for: First experience of hemadsorption with HA60 cartridges in newborns and small infants with septic shock: a case series
Source: Front Pediatr. 2026 Jun 5;14:1769731. doi: 10.3389/fped.2026.1769731 (PMC13279426; doi:10.3389/fped.2026.1769731)

**Supplementary File**

**Supplementary Figure 1.** Evolution of bowel perfusion investigated by echo-doppler ultrasonography before and after a cycle of HA60 hemoadsorption.


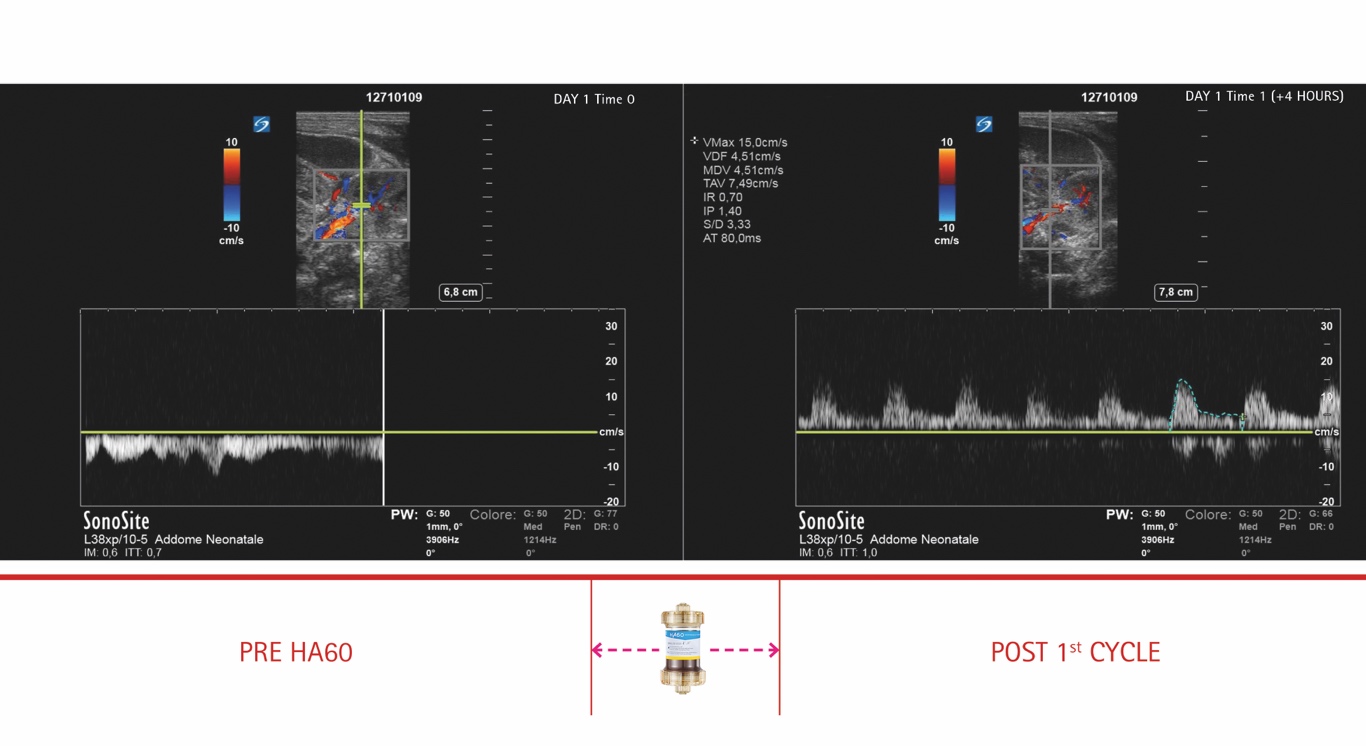


**Supplementary Figure 2.** Evolution of acute lung injury assessed by chest x-ray before and after 48 hours of HA60 hemoadsorption.


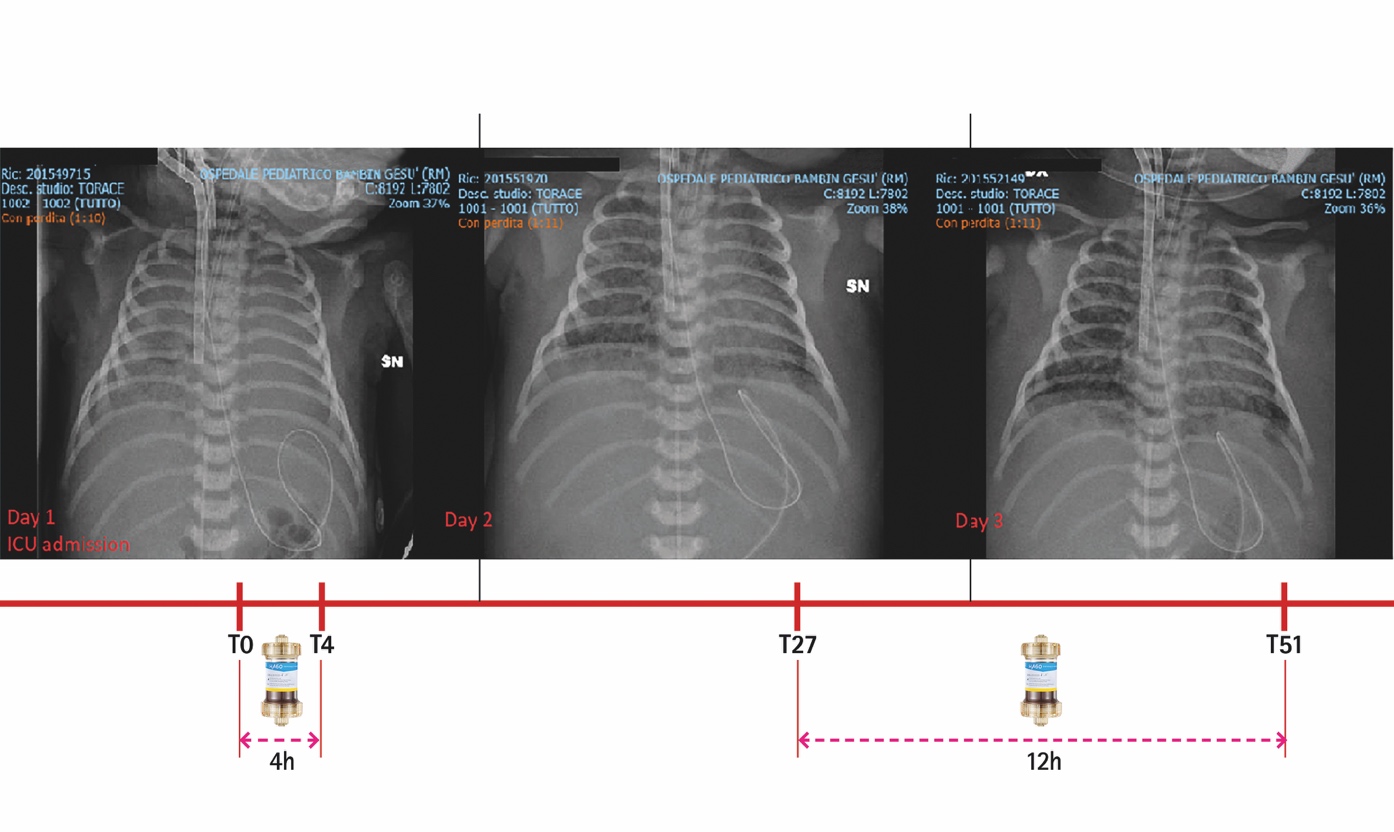

Supplement: Supplementary file 1 [file Supplementaryfile1.docx]
